# Higher Temperature at Lower Elevation Sites Fails to Promote Acclimation or Adaptation to Heat Stress During Pollen Germination

**DOI:** 10.3389/fpls.2018.00536

**Published:** 2018-04-30

**Authors:** Lluvia Flores-Rentería, Amy V. Whipple, Gilbert J. Benally, Adair Patterson, Brandon Canyon, Catherine A. Gehring

**Affiliations:** ^1^Department of Biology, San Diego State University, San Diego, CA, United States; ^2^Department of Biological Sciences and Merriam-Powell Center for Environmental Research, Northern Arizona University, Flagstaff, AZ, United States

**Keywords:** acclimation, elevational gradient, global climate change, heat stress, *Pinus edulis*, pollen development, pollen germination and viability, reproduction

## Abstract

High temperatures associated with climate change are expected to be detrimental for aspects of plant reproduction, such as pollen viability. We hypothesized that (1) higher peak temperatures predicted with climate change would have a minimal effect on pollen viability, while high temperatures during pollen germination would negatively affect pollen viability, (2) high temperatures during pollen dispersal would facilitate acclimation to high temperatures during pollen germination, and (3) pollen from populations at sites with warmer average temperatures would be better adapted to high temperature peaks. We tested these hypotheses in *Pinus edulis*, a species with demonstrated sensitivity to climate change, using populations along an elevational gradient. We tested for acclimation to high temperatures by measuring pollen viability during dispersal and germination stages in pollen subjected to 30, 35, and 40°C in a factorial design. We also characterized pollen phenology and measured pollen heat tolerance using trees from nine sites along a 200 m elevational gradient that varied 4°C in temperature. We demonstrated that this gradient is biologically meaningful by evaluating variation in vegetation composition and *P. edulis* performance. Male reproduction was negatively affected by high temperatures, with stronger effects during pollen germination than pollen dispersal. Populations along the elevational gradient varied in pollen phenology, vegetation composition, plant water stress, nutrient availability, and plant growth. In contrast to our hypothesis, pollen viability was highest in pinyons from mid-elevation sites rather than from lower elevation sites. We found no evidence of acclimation or adaptation of pollen to high temperatures. Maximal plant performance as measured by growth did not occur at the same elevation as maximal pollen viability. These results indicate that periods of high temperature negatively affected sexual reproduction, such that even high pollen production may not result in successful fertilization due to low germination. Acquired thermotolerance might not limit these impacts, but pinyon could avoid heat stress by phenological adjustment of pollen development. Higher pollen viability at the core of the distribution could be explained by an optimal combination of biotic and abiotic environmental factors. The disconnect between measures of growth and pollen production suggests that vigor metrics may not accurately estimate reproduction.

## Introduction

Extensive forest tree mortality associated with unusually dry and hot climatic conditions has been documented around the world (Allen et al., 2015). Physiological stress associated with changes in temperature and precipitation is expected to lead to tree mortality in the future (Allen et al., 2010; Anderegg et al., 2012). Species with small populations, fragmented geographic ranges, and/or low fecundity are likely to experience dramatic shifts in distribution (Aitken et al., 2008). Pinyon pine (*Pinus edulis*), in particular, is experiencing high mortality, low seed production, and low recruitment (Redmond et al., 2012; Redmond and Barger, 2013). Very little is known about the susceptibility of conifer pollen to heat stress in warm climates and whether there is the capacity for adaptation or acclimation to heat stress within populations.

Most predictions of the future distribution and conservation status of plants do not consider the effect of climate change on reproduction, even though studies of crop plants have shown that the sexual phase of plant reproduction, including pollen development, is a particularly vulnerable stage (Zinn et al., 2010). The effect of heat stress on pollen development is species dependent. Heat stress applied to the pollen donor in tomato, before and during pollen release, decreased seed and fruit set more than heat stress applied to the developing ovule and to the style after pollen application (Peet et al., 1998). In other species, heat stress had larger effects at later pollen developmental stages, particularly during the initiation of pollen tube tip growth and fertilization (Zinn et al., 2010, and the references therein). The only previous study of the effect of heat stress on pollen viability in conifers focused exclusively on pollen germination and found that pollen growth was reduced under heat stress (Frankis and Grayson, 1990). These limited data suggest that pollen development in forest trees might be vulnerable to heat stress under climate change and that studies should be performed at a range of developmental stages and in a range of species and climatic regions.

Pines are wind pollinated and form a cloud of pollen that is released from microstrobili (pollen-producing cones) during an approximately 2-week-long dispersal stage (Williams, 2010; Robledo-Arnuncio, 2011). Pollen remains viable during dispersal by dehydration (Fernando et al., 2005). Once pollen grains land on a pollination droplet (Gelbart and von Aderkas, 2002; Leslie, 2010), they rehydrate and a pollen tube grows toward the nucelle, a process referred to as pollen germination. Two consecutive mitotic divisions occur during pollen tube elongation to produce sperm nuclei (Fernando et al., 2005), a process that is highly sensitive to elevated temperature in crops and *Arabidopsis* (Saini et al., 1984; Jäger et al., 2008; Sakata et al., 2010).

Exposing pollen to heat stress at different developmental stages (e.g., pre-dispersal, dispersal, and germination) can be used to detect if the stages differ in susceptibility to heat stress and to test whether pollen can acclimate to heat during pollen dispersal (Senthil-Kumar et al., 2003; Natarajan and Kuehny, 2008; Bokszczanin et al., 2013). Such studies are limited in conifers. Acquired thermotolerance (Senthil-Kumar et al., 2003; Natarajan and Kuehny, 2008; Bokszczanin et al., 2013) or local adaptation could also develop over longer time spans when plant populations are exposed to different temperatures along elevational gradients. Acclimation of an individual could result in greater thermal tolerance of its pollen. Alternatively, variation in pollen heat tolerance could arise from natural selection (e.g., Aitken et al., 2008; Alberto et al., 2010). Clinal variation in traits across elevational gradients that result from acclimation or differential selection (Barton, 1999) could mitigate the negative effects of heat stress associated with climate change (Alberto et al., 2010, 2013).

Studies incorporating elevational variation in vegetative, phenological, and reproductive traits may allow the comparison of different plant components to warming and be key to predict the ability of tree populations to cope with climate change. Phenology varies with elevation in a number of systems, with responses resulting from both genetic and environmental factors (Silen, 1963; Schuster et al., 1989; Bonner, 2003; Giménez-Benavides et al., 2011). For example, in the alpine plant *Syringa vulgaris*, a temperature decrease of 1°C postpones the onset of flowering by 6.6 days, resulting in a gradient of flowering times of ∼4 days per 100 m elevation (Larcher, 2006). However, there are no studies evaluating phenology and temperature effects on pollen viability along an elevational gradient that simultaneously consider heat stress tolerance within multiple time scales of pollen development in a natural tree population.

We studied pinyon pine (*P. edulis*), a widely distributed foundation species of woodlands in the southwestern United States, where it is considered a barometer of climate change due to its high sensitivity to climate variation (Betancourt et al., 1993; Breshears et al., 2005; Gray et al., 2006; Adams et al., 2009). Recently, it has suffered among the highest rates of mortality of any tree species in the southwestern United States due to a combination of high temperatures, low precipitation, and bark beetles (Breshears et al., 2005; Mueller et al., 2005; Gitlin et al., 2006; McDowell et al., 2008; Adams et al., 2009). *P. edulis* thus may be particularly sensitive to the temperatures projected for southwestern North America which are expected to increase 3.5–4°C over the next 60–90 years (IPCC, 2007, 2014) and to heat waves or extreme temperature events which are projected to increase in intensity, frequency, and length (Meehl et al., 2007). In addition, temperature is the main limiting factor for successful reproduction in pine trees, including pollen development (Parantainen and Pulkkinen, 2002).

In this study, we examined whether the higher temperatures predicted with climate change affected pollen viability during dispersal and germination and whether pollen from lower elevation, historically warmer populations, was more tolerant of these higher temperatures by testing the following hypotheses. We hypothesized that high temperatures during pollen dispersal would have a minimal effect on pollen viability, while high temperatures during pollen germination would negatively affect pollen viability (Hypothesis 1). Because some plants show acquired thermotolerance when subjected to a heat acclimation pre-treatment, we hypothesized that high temperatures during pollen dispersal would precondition pollen grains to be more tolerant of high temperatures during pollen germination (Hypothesis 2). We hypothesized that because the short elevational gradient varies widely in temperature there will be (a) acclimation or adaptation of pollen; (b) variation in phenology of male strobili production; and (c) variation in vegetation composition and plant performance (Hypothesis 3). Specifically, we predicted that pollen from plants growing at lower elevations would be more tolerant of high temperatures than pollen from plants at higher elevations due to adaptation or acclimation. We also predicted that pollen from plants at higher temperature, lower elevation sites would mature earlier than pollen from plants at cooler, higher elevation sites. Furthermore, we demonstrate that this elevational gradient is biologically meaningful by evaluating variation in: (a) vegetation composition and (b) *P. edulis* performance, and further correlating those measures with pollen viability.

Our study contributes to the understanding of how environmental variation at microgeographic scales can lead to differential selection, an important aspect of the evolution of forest trees that is often neglected because long-distance pollen dispersal is thought to override selection at small scales (Richardson et al., 2014; Scotti et al., 2016). However, most gene flow in conifers is limited, with a long tail of rare, long-distance dispersal events (Burczyk et al., 1996, 2004; Schuster and Mitton, 2000; Lian et al., 2001; Robledo-Arnuncio et al., 2004; Robledo-Arnuncio and Gil, 2005) in which subpopulations can be detected within hundreds or even dozens of meters (Mitton et al., 1989, 1998). Our study of *P. edulis* populations occurred in a harsh, recently colonized environment, along a strong elevation gradient (4°C change in a little over 200 m), conditions in which selection might be particularly intense, resulting in acclimation or adaptation at microgeographic scales.

## Materials and Methods

### Field Site Characteristics

We tested our hypotheses during 2 years of pollen development near Sunset Crater National Monument in northern Arizona. An eruption that lasted for 200 years, beginning in 1064 AD, gave origin to the cinder soil in the region and pinyon pine has only recolonized the area in the last 800 years (Krutch, 1974). *P. edulis* growing in Sunset Crater National Monument experience severe soil water and nutrient deficits and significantly more negative xylem water potentials than pinyon pine populations growing in adjacent sandy-loam soil (Mopper et al., 1991b; Cobb et al., 1994). The recent deposition of the cinder fields and their highly stressful edaphic conditions result in a dynamic system with the potential for intense natural selection (Cobb et al., 1994). Such extreme environmental conditions are reflected in high levels of herbivory and mycorrhizal colonization (Whitham and Mopper, 1985; Gehring and Whitham, 1991, 1994; Mopper et al., 1991a,b; Del Vecchio et al., 1993) as well as slow growth (Mopper et al., 1991a) and low seed production, which has a negative impact on avian seed dispersal agents (Christensen and Whitham, 1991).

### Experiment 1 (Hypotheses 1 and 2)

#### Pollen Viability at One Elevational Point

We tested our first hypothesis that high temperatures during pollen dispersal would have a minimal effect on pollen viability, while high temperatures during pollen germination would negatively affect pollen viability, by exposing fresh pollen to four different temperatures representing current and predicted temperatures during pollen dispersal. The same experiment also allowed us to test our second hypothesis that high temperatures during pollen dispersal would acclimate pollen to be more tolerant of high temperatures during germination, expected by a positive interaction between the high-temperature treatments of the dispersal stage and germination stage.

**FIGURE 1:**
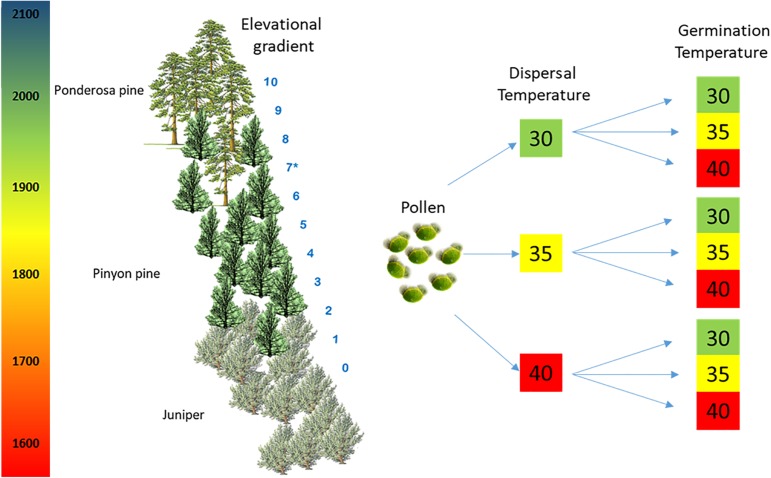
Experimental design. Experiment 1: Pollen from 24 trees was collected and warmed at different temperatures, pollen during the dispersal stage: 30, 35, 40, and 4°C which served as a control of optimal storage conditions (not shown in the figure). Reciprocal temperature treatments were also done during germination: 30, 35, and 40°C. This experiment was performed in 2012 from trees at the elevational gradient site 7^∗^. Experiment 2: Vegetation composition was determined at 11 sites along an elevational gradient (0–10). From sites 1–9, where pinyon pines were present, six trees per site were studied and pollen was warmed simulating the two temperature treatments, dispersal and germination, in a reciprocal fashion, as shown in the figure. Site 9 was excluded from the analysis because of its low pollen production. In the region of our study, there is a change in temperature of 4°C in a little over 200 m represented by the color gradient bar on the left, lower elevations experience higher temperature – red, compared to higher elevations – green.

Mature pollen was collected during the optimal pollen release time. Pollen collected in 2012 from 24 trees at 35°23.154 N, 111°26.331 W, 1902 m above sea level (site 7 in **Table 1** and **Figure 1**) was incubated in an oven for 1 week meant to represent the pollen dispersal stage, from anther dehiscence to pollen rehydration. Three incubation treatments and one control were performed: (a) 30°C which is the optimal germination temperature for *P. edulis* (Chira, 1965); this treatment matches the average maximum temperature (averaged arithmetic mean of all the daily high temperatures for the month) in the field during the time of pollen release, (b) 35°C which represents the averaged high temperature (the highest value recorded for June) recorded at the study site representing current heat waves, (c) 40°C which represents the estimated maximum future temperature, and (d) 4°C which served as a control of optimal storage conditions. We obtained ambient air temperatures from past weather (since 1940) recorded by NOAA http://www.nws.noaa.gov/ for Sunset Crater National Monument (higher elevation, Supplementary Table S1) and Wupatki National Monument (lower elevation, Supplementary Table S2) which border the distribution of *P. edulis* in our study site. Pollen from these four incubation treatments was divided into three germination treatments in a factorial design (30, 35, and 40°C). In order to assess pollen viability, pollen was placed in small petri dishes using the media 1% agar, 0.01% boric acid, and 2% sucrose to promote pollen germination, which includes pollen rehydration and subsequent pollen tube elongation (Chira, 1965). We determined that 4 days of incubation was the optimal time to assess the percentage of pollen viability as most of the viable pollen grains germinated at this time and pollen tubes were readily visible.

**Table 1:** Vegetation composition of the study sites based on the relative abundance of dominant tree species. Latitude and longitude coordinates and elevations are given for each site.

Site	Coordinates north	Coordinates west	Elevation (m.s.l.)^b^	Juniper:Pinyon: Ponderosa ratio
0^a^	35°25.441	111°23.322	1696	15:0:0
1	35°24.786	111°24.378	1748	5:8:0
2	35°24.239	111°25.121	1779	5:2:0
3	35°24.239	111°25.121	1800	0:3:0
4	35°23.860	111°25.506	1829	0:6:0
5	35°23.522	111°25.779	1860	0:14:0
6	35°23.428	111°25.303	1890	1:10:2
7	35°23.154	111°26.331	1902	0:8:5
8	35°22.804	111°26.631	1932	0:5:6
9^a^	35°22.617	111°27.274	1974	0:1:14
10^a^	35°22.30	111°27.343	2001	0:0:16


*^*a*^Sites with no pinyon pine (sites 0 and 10) or with very low pollen production (oxsite 9) were excluded from the pollen viability test. ^*b*^m.s.l. meters above sea level.*

**FIGURE 2:**
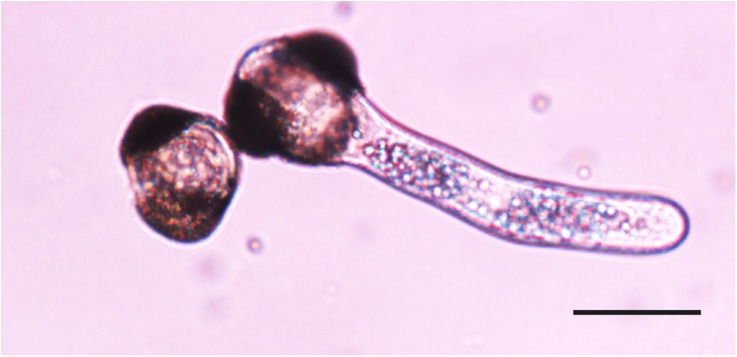
Viable pollen with pollen tube germinating (right) and non-viable pollen without pollen tube (left). Bar = 50 μm.

Pollen viability was evaluated by counting the number of germinated and non-germinated pollen grains in a single petri dish from each tree at each temperature using a dissecting microscope (Leica MZ6; **Figure 2**). All pollen grains visible at 2.5× magnification field were counted (average 147 pollen grains) and the percentage of germinated pollen grains was calculated. Pollen grains of 24 individuals were subjected to each of the four temperature treatments during pollen dispersal and subsequently to three temperature treatments during germination, for a total of 288 petri dishes analyzed in 2012.

### Experiment 2 (Hypothesis 3)

#### Vegetation, Phenology, and Plant Traits Across Elevation

First, we determined whether the short elevational gradient across which *P. edulis* is distributed at our study site has an effect on plant traits, as highlighted in studies evaluating the effects of abiotic factors on the life history traits variability at microgeographic scales (see Scotti et al., 2016). We measured (a) vegetation composition and (b) *P. edulis* performance-related life history traits. To evaluate changes in vegetation composition, we measured the relative abundance of dominant tree species in a quadrat of 50 m × 20 m. We selected 11 sites along a little over 300 m of elevational gradient, nine sites with *P. edulis* plus two adjacent sites in either lower and upper boundaries of its distribution range, capturing neighboring vegetation types (**Table 1** and **Figure 1**). Water stress is expected to be higher at lower elevations where trees are more exposed and temperatures are higher. We expected that trees that had less water stress or higher nutrient availability would grow larger. In order to evaluate the association between plant growth and water stress and/or nutrient availability, we measured shoot length as an indicator of growth from all of the trees from which we collected pollen, the ^13^C content of needles as an indicator of water stress, and needle C:N as an indicator of nutrient availability. Four mature needles were collected per tree, dried at 60°C to constant weight, and ground to powder using a 2000 geno grinder (SPEX Sample prep). The C and N content and isotopic composition of ground needles was analyzed by the Colorado Plateau Stable Isotope Laboratory (CPSIL)^1^ using a Carlo Erba NC 2,100 Elemental Analyzer (CE Instruments, Milan, Italy) interfaced with a Thermo-Finnigan Delta Plus XL (Thermo-Electron, Bremen, Germany) isotope ratio mass spectrometer (IRMS). The annual growth, in 2013, of 10 shoots per tree was measured to the nearest cm with calipers. Average shoot growth was calculated per tree.

On average, temperature decreases by 1.1°C per 200 m increase in elevation (Körner, 2007). However, based on climatic data from weather stations near our study site, the same elevation difference results in at least twice the temperature increase (Supplementary Tables S1, S2), a marked change that could strongly affect phenology. We evaluated the phenology of male strobili production and pollen development by devising six developmental categories ranging from no microstrobili initiation to microstrobili with complete pollen release. We counted the number of microstrobili per category for each of six trees at each of the nine elevations. Because pollen development within a tree is highly synchronous, we assigned each tree to a category of pollen development if 80% or more of its microstrobili were in the same developmental category. To confirm the temperature gradient predicted based on the elevation gradient, we measured the temperature around stem tips where microstrobili and megastrobili occur. We placed three temperature loggers (Onset TidbiT v2 Temp Data Logger) per site at the lowest, middle, and highest elevation sites and recorded temperature at 15 min intervals for 6 days during pollen dispersal. Data from the three loggers per site were averaged to obtain both average 24 h temperature, and average daytime and nighttime temperatures experienced by microstrobili at each site.

#### Pollen Viability Across an Elevational Gradient

We tested our hypothesis that pollen viability would be strongly associated with temperature variation among elevations by evaluating pollen viability (**Figure 1**). In 2013, we collected pollen from the nine elevational points encompassing the pinyon pine distribution at the study site. However, samples from the highest elevation (1974 m) were excluded from pollen viability measurements because trees at this site produced insufficient pollen for accurate measurement of viability (**Table 1**). We used the same methods as described for experiment 1, except that we included pollen source site along the elevational gradient as an additional factor. We processed pollen as described above except we omitted the fourth treatment (control under optimal storage conditions, 4°C). The experiment was thus a fully factorial design including pollen source location (eight sites), pollen dispersal temperature (30, 35, and 40°C), and pollen germination temperature (30, 35, and 40°C). As in experiment 1, mature pollen was collected during optimal pollen release.

### Statistical Analyses

We conducted logistic regression for both pollen germination experiments using the generalized linear model command in R (R Core Team, 2014) with binomial family functions. Pollen dispersal and germination temperatures were included as factors and two-way interactions for dispersal and germination temperatures were included in the models. Three-way interactions were included for the elevation source experiment with elevation included as an ordered factor because the distribution of collection points was scattered. Linear and squared terms for the elevation predictive variable were included in the model.

Clinal morphological and physiological patterns are better described by the slope of a regression along an environmental gradient such as elevation (Alberto et al., 2013). A linear regression was performed in JMP Pro 11 (JMP^®^, 1989–2007) to analyze the effect of elevation on field pollen phenology, shoot growth, ^13^C, and C:N.

## Results

### Pollen Viability at One Elevational Point

**FIGURE 3:**
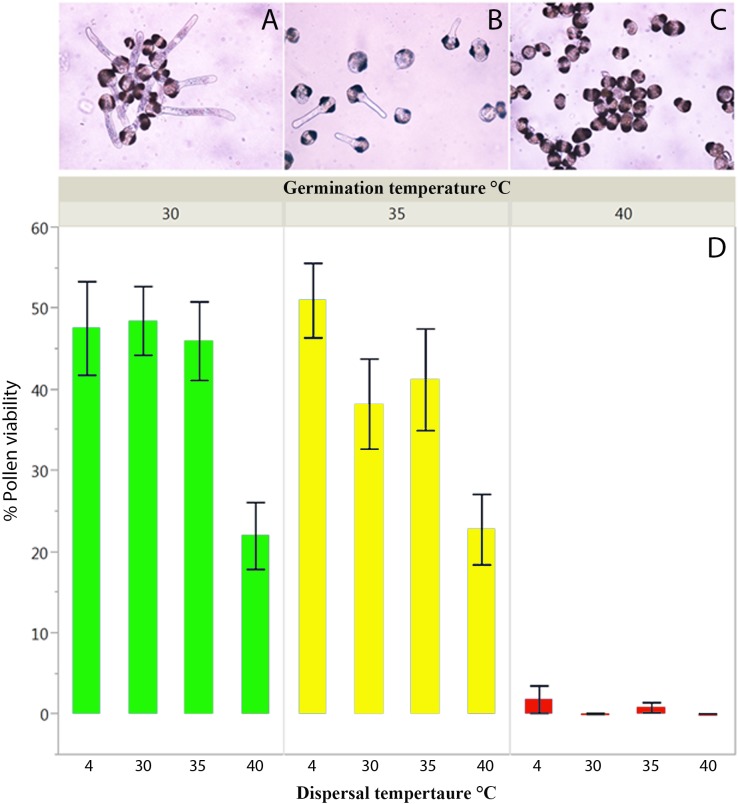
Pollen viability/germinability from 2012 from trees at 2010 m of elevation experimentally subjected to **(A)** 30, **(B)** 35, and **(C)** 40°C. **(D)** Mean percentage pollen germination when pollen was incubated at 4, 30, 35, and 40°C during pollen dispersal as a precondition treatment and reciprocally germinated at 30 (green), 35 (yellow), and 40°C (red). Error bars represent one standard error from the mean.

We found that pollen viability was affected by high temperatures at both dispersal and germination stages of pollen development. During dispersal, pollen was susceptible to increased temperatures, in particular when incubated at 40°C (GLM, _Z3,279_ = -19.230, *P* < 0.0001). However, heat stress had a larger effect during germination than during dispersal as inferred by the higher deviance of germination explaining the majority of variance in the model (**Table 2**). Pollen germination was similar in the 30 and 35°C treatments, but dropped significantly at 40°C (GLM, _Z2,277_ = -28.512, *P* < 0.0001). Regardless of the temperature at dispersal, pollen viability was close to zero when germination occurred at 40°C (**Figure 3C** – extreme right and **Figure 3D** – red bars). In addition to the effects of heat stress on pollen viability, cellular differences were detected among pollen grains incubated at different temperatures during germination. The pollen tubes germinated at 30°C (**Figure 3A**) show denser cytoplasmic (e.g., starch) content in comparison to the pollen germinated at 35 (**Figure 3B**) and 40°C (**Figure 3C**).

**Table 2:** Statistical estimates for the generalized linear model including the incubation at dispersal (DS) and germination (GS) stages of development as well as the two-way interactions.

	Df	Deviance	Resid. Df	Resid. Dev	Pr(>Chi)	
DS	3	1049.8	279	17164.4	<2.2e-16	^∗∗∗^
GS	2	8901	277	8263.4	<2.2e-16	^∗∗∗^
DS:GS	6	170.6	271	8092.8	<2.2e-16	^∗∗∗^


*Deviance of GS explained the majority of variance in the model. Signif. codes: “^∗∗∗^”, 0; “^∗∗^”, 0.001; “^∗^”, 0.01.*

We found a significant negative interaction between pollen viability at dispersal and germination (**Table 2** and Supplementary Table S3). The incubation of 30 and 35°C and the control at 4°C of pollen at dispersal showed similar viability when the germination treatment was done at 30°C, yielding approximately 48% viability. A sudden decrease was detected with 40°C at dispersal, in which pollen viability dropped by more than half to 22% (**Figure 3D**). Although there was a tendency toward lower pollen viability with germination incubation of 35°C, in particular when dispersal occurred at 30°C, the patterns of pollen viability at 30 and 35°C during germination were similar, and both show an important reduction of pollen viability when dispersal occurs at 40°C.

### Vegetation, Phenology, and Plant Traits Across Elevation

**FIGURE 4:**
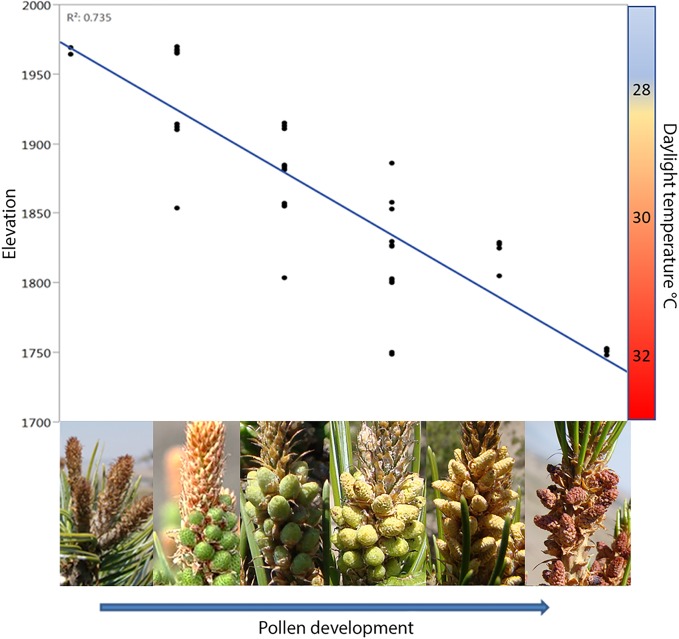
The phase of pollen development observed in *P. edulis* at June 1 2013 across an elevational gradient near Sunset Crater Arizona. There is a negative relationship between pollen development and elevation. Temperature difference between low and high elevation in the elevational gradient for *P. edulis* was 4°C, with 28, 30, and 32°C for high, middle, and low elevation, respectively, when considering only daylight temperatures.

Our measurements of the temperature around microstrobili and megastrobili during pollination along the elevational gradient showed that temperatures varied as much as 4°C (Supplementary Figure S2). Daytime temperatures averaged 28, 30, and 32°C for high, middle, and low elevation, respectively (**Figure 4**). Pollen development time was negatively, linearly associated with increasing elevation (*R*^2^ = 0.735, *F* = 113.48, *P* < 0.0001, **Figure 4**), supporting the hypothesis that the timing of pollen development is strongly associated with elevation and in particular that pollen from plants at higher temperature, lower elevation sites would mature earlier than pollen from plants at cooler, higher elevation sites. At the time when lower elevation trees (∼1750 msl) had completely released their pollen, higher elevations trees (>1950) had either just begun to develop microstrobili or had not yet produced microstrobili primordia. Approximately 2 weeks were required to complete development from microstrobili formation to full pollen release at all sites.

**FIGURE 5:**
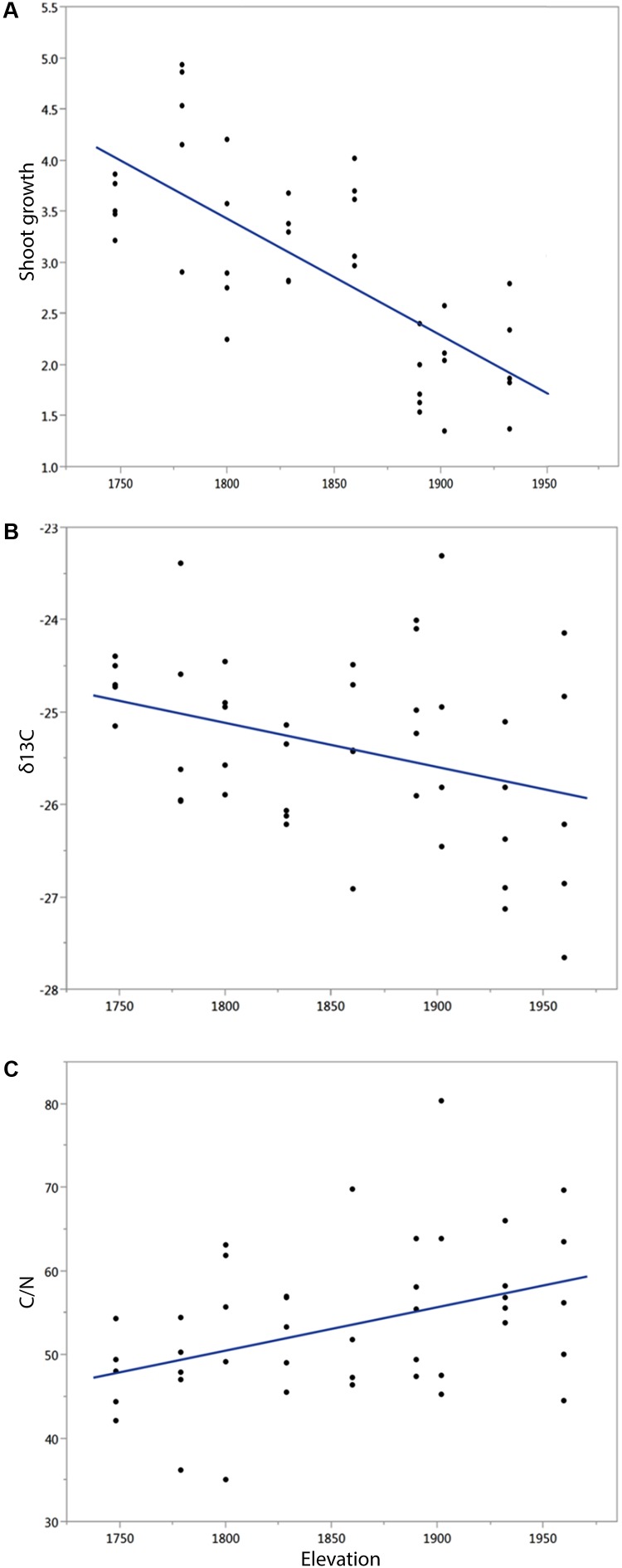
Elevation correlates negatively with **(A)** average shoot growth (cm) of *Pinus edulis* and **(B)** water stress inferred by isotopic δC^13^ preference. **(C)** Elevation correlates positively with C:N ratio. Each point represents the data for a single tree.

The dominant woody vegetation also differed with elevation. The lowest elevation site (0) showed extreme dominance by *Juniperus monosperma* with only an occasional pinyon, while sites 1 and 2 were mixed juniper and pinyon, sites 3–5 were pinyon dominated, sites 6–8 were a mixture of pinyon and ponderosa pine, and sites 9 and 10 were dominated by ponderosa pine (**Table 1**). Shoot growth was linearly and negatively correlated with elevation (*R*^2^ = 0.535, *F* = 43.6873, *P* < 0.0001, **Figure 5A**), and δ^13^C needle content (*R*^2^ = 0.118, *F* = 5.6, *P* = 0.0227, **Figure 5B**), less negative values represent less carbon isotope discrimination against ^13^C which indicates more water stress. In contrast, C:N content was linearly and positively correlated with elevation (*R*^2^ = 0.165, *F* = 8.29, *P* = 0.0062, **Figure 5C**).

### Pollen Viability Across an Elevational Gradient

**FIGURE 6:**
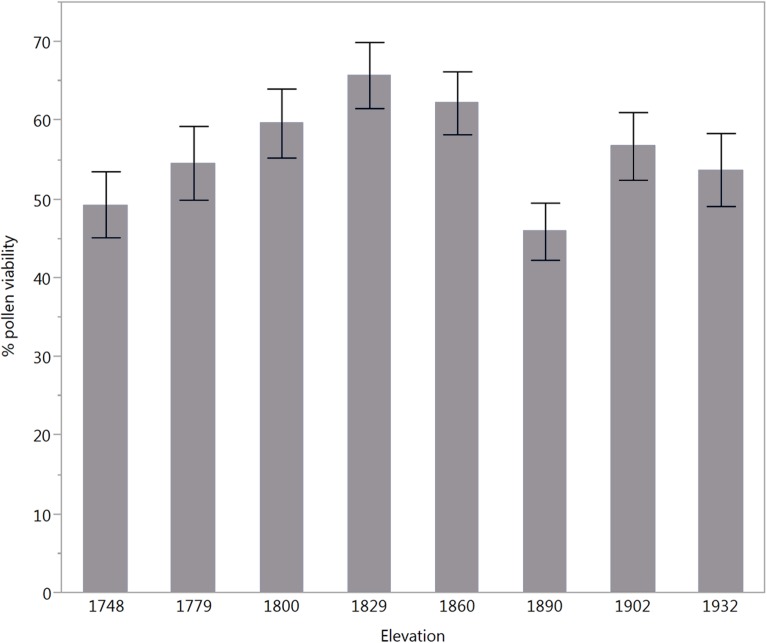
Quadratic distribution of pollen viability/germinability of *P. edulis* along an elevational gradient showing higher pollen germination from sites at middle elevation.

The hypothesis that pollen viability would be strongly associated with elevation was supported (**Figure 6** and **Table 3**). Pollen viability showed a quadratic distribution with elevation where lower elevation sites (1748 and 1779 m) and high elevation sites (1902 and 1932 m) had lower average viability than middle elevation sites (1800–1860 m; **Figure 6**). This result is supported by the significance of the quadratic term of elevation but not the linear term (**Table 3**). However, our results do not support the prediction that pollen from plants growing at lower elevations is more tolerant of higher temperatures than pollen from plants at higher elevations (Supplementary Figure S3 and Supplementary Table S4).

**Table 3:** Statistical estimates for the generalized linear model including the temperature at the dispersal stage (DS), germination stage (GS), and the elevational components as well as the two-way and three-way interactions.

	Df	Deviance	Resid. Df	Resid. Dev	Pr(>Chi)	
DS	2	50.9	357	15707.5	9.02E-12	^∗∗∗^
GS	2	9870.3	355	5837.2	<2.2e-16	^∗∗∗^
Alt1	1	0.5	354	5836.7	0.46853	
Alt2	1	259.6	353	5577.1	<2.2e-16	^∗∗∗^
DS:GS	4	106.1	349	5471	<2.2e-16	^∗∗∗^
DS:Alt1	2	34.7	347	5436.3	2.93E-08	^∗∗∗^
GS:Alt1	2	108.6	345	5327.7	<2.2e-16	^∗∗∗^
DS:Alt2	2	4.2	343	5323.5	0.12518	
GS:Alt2	2	8.7	341	5314.8	0.01274	^∗^
DS:GS:Alt1	4	90.7	337	5224.1	<2.2e-16	^∗∗∗^
DS:GS:Alt2	4	83.4	333	5140.7	<2.2e-16	^∗∗∗^


*Alt1and Alt2 represent the linear and quadratic term, respectively. Signif. codes: “^∗∗∗^”, 0; “^∗∗^”, 0.001; “^∗^”, 0.01.*

**FIGURE 7:**
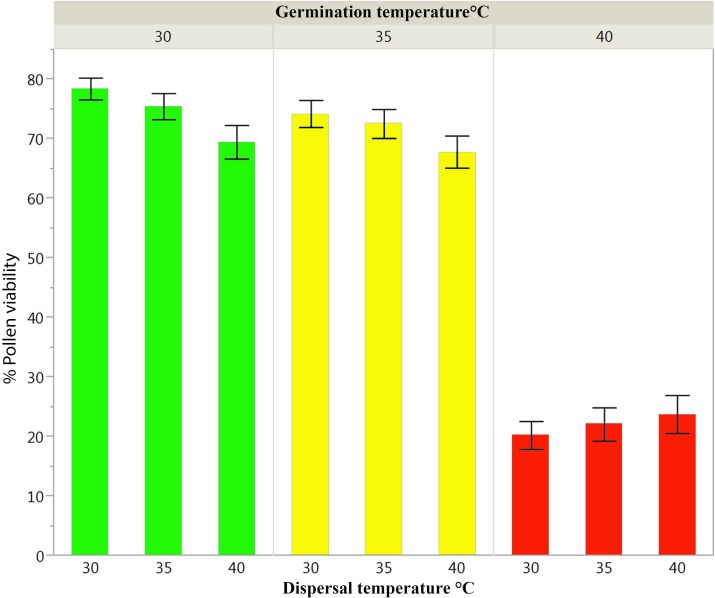
Mean percentage viability of pollen incubated at 30, 35, and 40°C at dispersal and reciprocally germinated at 30 (green), 35 (yellow), and 40°C (red) along the elevational gradient using all sites combined. Error bars were constructed using one standard error from the mean.

The overall pollen viability at dispersal and germination along the elevational gradient in 2013 was higher than the single elevational point in 2012 in all of the treatments; however, the results are qualitatively similar across years. Consistent with the 2012 data, there was (a) a significant effect of temperature on both the dispersal and germination phases of pollen development with increasing temperatures negatively affecting pollen viability, (b) an interaction between pollen viability at dispersal and germination (**Table 3**), and (c) a drop in pollen viability during germination at 40°C (**Figure 7**). The significant three-way interaction among pollen viability at dispersal, germination, and elevation in the linear and quadratic terms (**Table 3**) indicates that heat stress had an effect on pollen viability during both dispersal and germination stages and that the magnitude of this effect varied with elevation.

## Discussion

### Predicted Temperatures Under Climate Change Affect Male Reproduction

Viability during pollen dispersal and germination in *P. edulis* was negatively affected by the warmer temperatures predicted for the next century. At release, pine pollen is dehydrated and in a state of reduced metabolic activity, allowing dispersal over greater distances, longer survival, and greater resistance to low relative humidity (Fernando et al., 2005; Franchi et al., 2011; Firon et al., 2012). We thus hypothesized that temperature would not have a significant effect on pollen during this stage, but found that *P. edulis* pollen was susceptible to high temperatures even during this “protected” stage. The sensitivity of *P. edulis* during the dispersal stage is particularly surprising given its distribution in the warm, semi-arid southwestern United States, and previous work on *Pinus taeda* in which pollen viability remained close to 100% even at 37°C (Bohrerova et al., 2009). Pollen germination at 40°C was near zero in both experiments, supporting our hypothesis that the germination stage would be more negatively affected by high temperatures. Pollen elongation may be a more susceptible developmental stage because the RNA necessary for pollen germination and early tube growth is present before germination, while tube elongation depends on continuous protein synthesis (Hao et al., 2005).

*Pinus edulis* is likely to already have experienced the 40°C estimated maximum temperature under climate change used in our studies. Mean canopy leaf temperatures, through the absorption of radiant energy, can be up to 20°C higher than the surrounding air (Ansari and Loomis, 1959; Leuzinger and Köner, 2007; Leuzinger et al., 2010), and in evergreen conifers, even under winter conditions, this difference can be 15°C (Ehlers, 1915; Christersson and Sandstedt, 1978). Reproductive structures occurring at the tips of branches experience greater light intensity and can retain more heat than less exposed vegetative parts (Dietrich and Körner, 2014). Although we did not measure internal temperatures in the plant, our results support the negative effect of high predicted temperatures under climate change on pollen viability. Furthermore, our data suggest that if heatwaves are maintained during both the dispersal and germination phases, there will be little viable pollen available for reproduction (less than 1% in some localities and up to 20% for the whole gradient). Regardless of the actual temperature at which pollen becomes inviable in nature, our study suggests that an increment of a few degrees Celsius could have a negative impact on reproduction.

### High Temperature During Dispersal Does Not Acclimate Pollen to Resist High Temperatures During Germination

Pollen incubation at high temperatures during the lab-mimicked dispersal stage does not precondition pollen to resist high temperatures during germination as we hypothesized; in fact, there was a cumulative negative effect as shown by the dispersal by germination interaction. Thermotolerance can be achieved by modulating the expression levels of “responsive” or “protective” genes prior to heat stress exposure (Larkindale et al., 2005; Frank et al., 2009). Preconditioning to high temperature exposure might be more effective during earlier developmental stages such as microsporogenesis and microgametogenesis. During these stages, chromatin remodeling and nucleosome composition can be altered by high temperatures (Bokszczanin et al., 2013). In some cases, we detected aberrant pollen, possibly the result of odd meiotic processes that did not render the pollen inviable (Supplementary Figure S1). Aberrant meiosis has been reported in pinyon pines and other conifers as a result of extreme temperatures (Chira, 1965; Andersson, 2009). In wheat, abnormal pollen development can be induced by high temperature stress; the pollen mother cells complete meiosis but do not undergo mitosis (Saini et al., 1984), microspores do not have cytoplasm, and may remain immature. Although the cytoplasmic content, particularly starch content in the pollen tubes, was not quantified in our study, it was evident that cytoplasmic content was more abundant in the pollen germinated at 30°C than in the pollen germinated at 35 or 40°C. Plants with pollen tubes that are starch-limited could have reduced fertilization (Clément et al., 1994).

### Variation in Pollen Development and Pollen Viability Across an Elevational Gradient With Contrasting Environmental Attributes

Consistent with our predictions, pollen from *P. edulis* growing at higher temperatures and lower elevation sites developed earlier than pollen from trees at higher elevation. Asynchrony in strobili production along elevational transects has been described in conifers (Alberto et al., 2013). Changes in reproductive phenology (e.g., advancement of flowering time) are one of the most sensitive plant responses to warming (Wolkovich et al., 2012), and in some plant species, the negative correlation between elevation and temperature results in a gradient of flowering times of ∼4 days per 100 m elevation (Larcher, 2006). A similar pattern was observed in our >200 m elevational gradient in which microstrobili production and therefore pollen release was postponed for more than 8 days at the highest elevation compared to the lowest elevation site in the study area. Temperature thresholds for flowering time are genetically determined, well established, and may vary intraspecifically (Hänninen and Tanino, 2011). Alternatively, a plastic response could explain the shift in phenology along the elevational gradient. Advancing phenology in warmer temperatures at lower elevations could be an “escape strategy” to avoid warmer, later season temperatures (Gugger et al., 2015).

We expected that *P. edulis* trees that experienced higher mean temperatures (lower elevation) would be more tolerant of the higher temperatures predicted by climate models due to a combination of acclimation and adaptation. We observed higher pollen viability occurring in *P. edulis* from middle elevation sites (core of its distribution) than high and low sites (the edges of its distribution). Higher pollen viability at the core distribution remained the same at high and low germination temperatures, suggesting constant thermal tolerance across the elevation gradient. These findings support the idea that populations at intermediate elevations are under optimal environmental conditions, whereas the peripheral populations are in suboptimal situations (Ohsawa and Ide, 2008). An optimal combination of biotic and abiotic environmental conditions at the core of the distribution could be associated with greater pollen viability. For instance, resource competition with juniper at lower elevations and with ponderosa pine at upper elevations could reduce nutrient availability in the edges of pinyon pine distribution, resulting in lower pollen viability.

### Variation of Vegetation Composition and Plant Performance in a Short Elevational Gradient With Contrasting Environmental Attributes

We observed marked changes in vegetation composition across elevation, further supporting the idea of extreme environmental changes in a short elevational gradient. In other sites with *P. edulis*, its range of distribution can expand along 600 m in elevation (Trombulak and Cody, 1980). In our study site, *P. edulis* distributes along a little over 200 m elevation range, with a short transition to drier vegetation (juniper dominated) in lower elevation and to more mesic vegetation (ponderosa pine dominated) toward higher elevation. This dramatic change in vegetation could be attributed to the rapid temperature increase with elevation.

The δ^13^C data showed a negative linear association with elevation suggesting trees in lower elevations have higher water stress. Water use efficiency increases due to stomatal closure (reducing transpiration and less ^13^C discrimination) in stressful conditions (Farquhar and Richards, 1984). Concordantly, other studies in the southwest United States have shown that in C3 plants, including *P. edulis*, δ^13^C leaves have less discrimination at the lowest elevations, with extreme values at sites that receive more than 550 mm mean annual precipitation, the opposite of trends detected in more humid regions (Van de Water et al., 2002). In contrast, needle C:N ratio showed a positive linear relationship with elevation suggesting more nitrogen (N) availability in lower elevations. In environments with low N availability, plants often fix more carbon and produce more biomass per unit of N (i.e., they have a high N use efficiency, Vitousek, 1982; Aerts and Chapin, 2000), which is consistent with our results. Moreover, the C:N ratios of forest soils are tree species dependent (Cools et al., 2014), because our elevational gradient encompasses three types of vegetation (junipers, pinyon pine, and ponderosa pine dominated), changes in the C:N ratio might also be associated with changes in the dominant species. The higher stress observed at lower elevations and the low nitrogen availability in upper elevations might contribute to the quadratic response of pollen viability on the elevational gradient. However, growth, which could also be subjected to this tradeoff, has a negative linear relationship with elevation.

## Conclusion

Our findings indicate that pollen viability may decrease with increased temperatures predicted under climate change, potentially reducing reproduction in *P. edulis.* Although increased dispersal, seed production (Kellomäki et al., 1997), and improved germination and establishment are expected as result of the warmer air and soil in some species (Kuparinen et al., 2009; Alberto et al., 2013), the opposite may be true for *P. edulis* and other species which inhabit hotter and more arid environments. Redmond et al. (2012) documented declines in pinyon pine cone production with higher temperatures, with the greatest negative effects observed in populations experiencing the largest increases in temperature. Low reproduction in *P. edulis* could have a tremendous impact on southwestern US forest, where pinyon pine is a foundation species that associates with a diverse community of microbes, insects, and vertebrates (Whitham et al., 2003). *P. edulis* has suffered among the highest rates of mortality of any tree species in the southwestern United States due to climate change (Breshears et al., 2005; Mueller et al., 2005; Gitlin et al., 2006; McDowell et al., 2008; Adams et al., 2009). Our findings add a new dimension to our understanding of the consequences of climate change by showing that male reproduction is negatively affected by high temperatures. The pollen of *P. edulis* may be particularly sensitive to the increase of 3.5–4°C predicted over the next 60–90 years based on the moderate A1B carbon-emission scenario (IPCC, 2014), or with projected increased heat waves or extreme temperature events (Meehl et al., 2007). Poor reproduction could hamper the recovery of *P. edulis* populations following high mortality events, but high mortality sites could also represent establishment opportunities for better adapted genotypes generated by gene flow and local selection (Kuparinen et al., 2010). However, strong selection operating on pinyon pine pollen viability might not result in rapid loss of heat susceptible genotypes because vegetative growth and water use efficiency show opposing patterns with warming compared to pollen development. Climate models predict that the range of *P. edulis* could contract substantially with continued warming (e.g., Rehfeldt et al., 2006; Cole et al., 2007) and our study provides evidence that lower reproductive success could contribute importantly to this change.

## Author Contributions

LF-R, CG, and AW designed the study. All authors collected samples and field data. LF-R, GB, and BC estimated pollen viability. LF-R prepared samples for isotope analysis. LF-R and AW analyzed the data. LF-R, AW, and CG wrote the manuscript. All authors contributed to revisions.

## Conflict of Interest Statement

The authors declare that the research was conducted in the absence of any commercial or financial relationships that could be construed as a potential conflict of interest.

## Footnotes

1 http://www.isotope.nau.edu
