# Supplementary material for: Higher Temperature at Lower Elevation Sites Fails to Promote Acclimation or Adaptation to Heat Stress During Pollen Germination
Source: Front Plant Sci. 2018 Apr 30;9:536. doi: 10.3389/fpls.2018.00536 (PMC5936790; doi:10.3389/fpls.2018.00536)
Supplement: Supplementary file 1 [file Table_1.DOCX]

SUPPLEMENTARY DATA


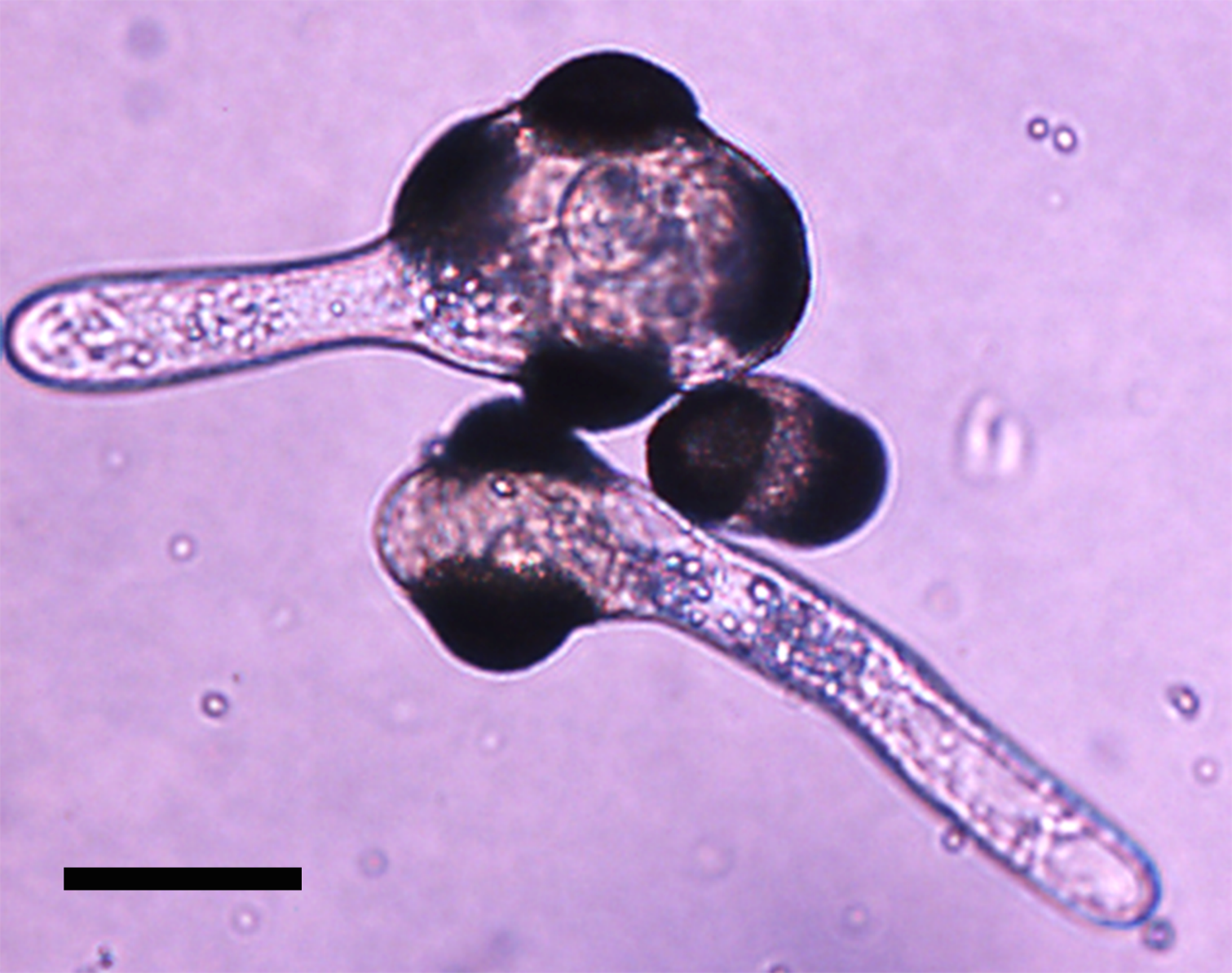


Fig S1. Abnormal pollen grain with four pollen sacci (top) with apparent pollen tube development and pollen grain with two sacci with pollen tube. Bars=80 µM.

Fig S2. Averaged temperature along the altitudinal gradient. We selected the lowest, middle and highest elevations of the elevational gradient at our study site and placed three temperature loggers per site, each set to measure temperature at 15 min intervals for 6 days. Average temperatures of three loggers per site were averaged calculating temperature differences among sites for the whole recording (24 hours/6 days). Lower, middle and higher elevation correspond to 1748, 1850 and 1904 m.s.l. respectively.


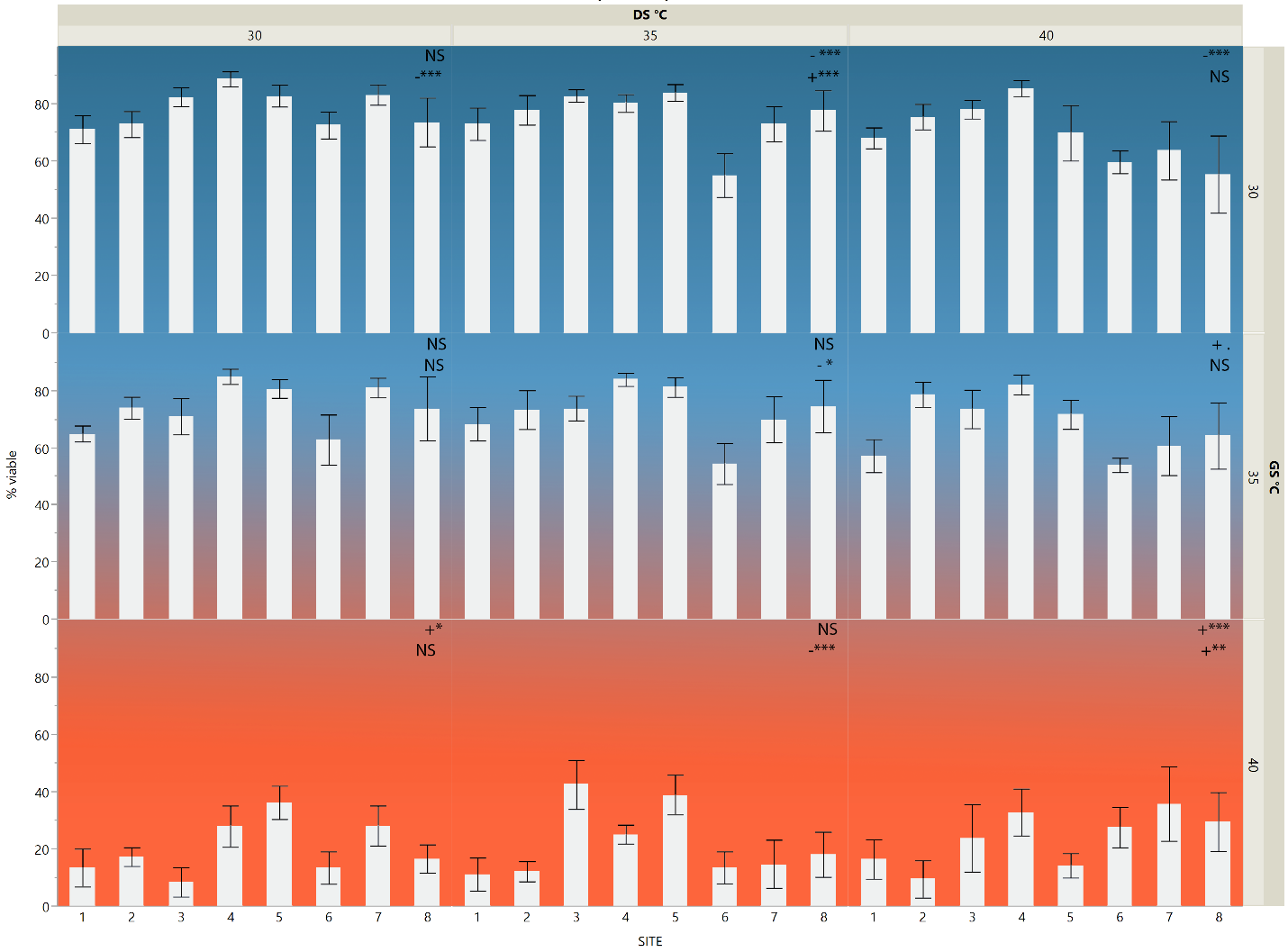


Fig S3. Bar plots showing mean percentage pollen viability by combined treatments: dispersal stage temperature (DS) by germination stage temperature (GS) by eight altitudes. Pollen preconditioned to heat stress during DS was subjected to 30 °C, 35 °C and 40 °C and reciprocally germinated (GS) at 30 °C, 35 °C and 40 °C along the altitudinal gradient. Statistical estimates for the generalized linear model including the temperature at the dispersal stage (DS), germination stage (GS) and the altitudinal components as well as the two way and three way interactions are shown in Table 2S. Significance of the linear and quadratic terms are shown on the right-up corner of each panel. Signif. codes: NS ‘non significant’ 0 ‘***’ 0.001 ‘**’ 0.01 ‘*’

Table S1. Historical climatic data from Sunset Crater National Monument, Arizona (weather station number: 028329). Period of Record: 12/01/1969 to 06/10/2016. Average maximum temperature of 27.72 C (81.9 F) in June at Sunset Crater National Monument, Arizona = 2127 m/ 6980 ft. Historical high temperatures taken from two weather stations (<http://www.wrcc.dri.edu/>).

|  | Jan | Feb | Mar | Apr | May | Jun | Jul | Aug | Sep | Oct | Nov | Dec | Annual |
| --- | --- | --- | --- | --- | --- | --- | --- | --- | --- | --- | --- | --- | --- |
| Average Max. Temperature (F) | 43.8 | 46.5 | 53.1 | 61.2 | 70.8 | 81.9 | 84.5 | 81.3 | 75.4 | 64.2 | 52.4 | 44.4 | 63.3 |
| Average Min. Temperature (F) | 12.3 | 15.9 | 20.8 | 26.1 | 33.2 | 40.4 | 48.0 | 46.4 | 38.9 | 27.8 | 18.7 | 12.4 | 28.4 |
| Average Total Precipitation (in.) | 1.30 | 1.21 | 1.21 | 0.74 | 0.68 | 0.42 | 2.49 | 2.99 | 1.84 | 1.36 | 1.14 | 1.42 | 16.79 |
| Average Total SnowFall (in.) | 14.1 | 11.3 | 10.1 | 3.6 | 0.4 | 0.0 | 0.0 | 0.0 | 0.0 | 1.4 | 5.3 | 13.3 | 59.5 |
| Average Snow Depth (in.) | 5 | 4 | 2 | 0 | 0 | 0 | 0 | 0 | 0 | 0 | 0 | 3 | 1 |

Table S2. Historical climatic data from Wupatki National Monument, Arizona (weather station number: 029542). Period of Record: 7/ 1/1948 to 12/31/2005. Average maximum temperature of 33.22 (91.8) C in June at Wupatki National Monument, Arizona = 1554.691 m / 5100.691 ft. Historical high temperatures taken from two weather stations (<http://www.wrcc.dri.edu/>).

|  | Jan | Feb | Mar | Apr | May | Jun | Jul | Aug | Sep | Oct | Nov | Dec | Annual |
| --- | --- | --- | --- | --- | --- | --- | --- | --- | --- | --- | --- | --- | --- |
| Average Max. Temperature (F) | 47.3 | 54.9 | 62.7 | 71.6 | 81.2 | 91.9 | 95.4 | 92.0 | 85.8 | 73.6 | 58.3 | 47.0 | 71.8 |
| Average Min. Temperature (F) | 24.5 | 29.0 | 34.5 | 41.5 | 50.0 | 59.6 | 65.6 | 63.1 | 56.4 | 45.2 | 33.2 | 24.7 | 43.9 |
| Average Total Precipitation (in.) | 0.44 | 0.43 | 0.61 | 0.38 | 0.35 | 0.30 | 1.37 | 1.58 | 0.93 | 0.70 | 0.52 | 0.49 | 8.11 |
| Average Total SnowFall (in.) | 1.4 | 1.0 | 1.2 | 0.2 | 0.0 | 0.0 | 0.0 | 0.0 | 0.0 | 0.0 | 0.5 | 2.2 | 6.4 |
| Average Snow Depth (in.) | 0 | 0 | 0 | 0 | 0 | 0 | 0 | 0 | 0 | 0 | 0 | 0 | 0 |

Table S3. Complete output for the statistical GLM of the pollen viability with coefficients for pollen viability for data on 2012. Statistical estimates for the generalized linear model including the incubation (DS) and germination (GS) components as well as the two way interactions. Signif. codes: 0 ‘***’ 0.001 ‘**’ 0.01 ‘*’.

|  | Estimate | Std. Error | z value | Pr(>\|z\|) |  |
| --- | --- | --- | --- | --- | --- |
| (Intercept) | -0.09647 | 0.03412 | -2.828 | 0.00469 | ** |
| DS30 | 0.06794 | 0.04904 | 1.385 | 0.16597 |  |
| DS35 | -0.03729 | 0.04802 | -0.776 | 0.43748 |  |
| DS40 | -1.01891 | 0.05299 | -19.23 | < 2e-16 | *** |
| GS35 | 0.13521 | 0.04568 | 2.96 | 0.00308 | ** |
| GS40 | -3.68046 | 0.12908 | -28.512 | < 2e-16 | *** |
| DS30:GS35 | -0.58275 | 0.06701 | -8.696 | < 2e-16 | *** |
| DS35:GS35 | -0.35488 | 0.06599 | -5.378 | 7.53E-08 | *** |
| DS40:GS35 | -0.10824 | 0.07319 | -1.479 | 0.13913 |  |
| DS30:GS40 | -3.52986 | 0.7199 | -4.903 | 9.43E-07 | *** |
| DS35:GS40 | -0.6963 | 0.22226 | -3.133 | 0.00173 | ** |
| DS40:GS40 | -14.7659 | 196.2793 | -0.075 | 0.94003 |  |

Table S4. Complete output for the statistical GLM of the pollen viability with coefficients for pollen viability for data on 2014. Statistical estimates for the generalized linear model including the incubation (DS), germination (GS) and altitude (linear *Alt1* and quadratic *Alt2* terms) components as well as the two and three way interactions. Signif. codes: 0 ‘***’ 0.001 ‘**’ 0.01 ‘*’.

|  | Estimate | Std. Error | z value | Pr(>\|z\|) |  |
| --- | --- | --- | --- | --- | --- |
| (Intercept) | 1.29605 | 0.03654 | 35.471 | < 2e-16 | *** |
| DS35 | -0.18459 | 0.04972 | -3.713 | 0.000205 | *** |
| DS40 | -0.44927 | 0.04908 | -9.154 | < 2e-16 | *** |
| GS35 | -0.23935 | 0.04966 | -4.82 | 1.44E-06 | *** |
| GS40 | -2.68445 | 0.0524 | -51.228 | < 2e-16 | *** |
| Alt1 | 1.03497 | 0.64756 | 1.598 | 0.109986 |  |
| Alt2 | -4.60043 | 0.6668 | -6.899 | 5.23E-12 | *** |
| DS35:GS35 | 0.09771 | 0.06849 | 1.427 | 0.153661 |  |
| DS40:GS35 | 0.13769 | 0.06737 | 2.044 | 0.040977 | * |
| DS35:GS40 | 0.26561 | 0.07291 | 3.643 | 0.000269 | *** |
| DS40:GS40 | 0.6504 | 0.07134 | 9.116 | < 2e-16 | *** |
| DS35:Alt1 | -3.11432 | 0.90595 | -3.438 | 0.000587 | *** |
| DS40:Alt1 | -6.08486 | 0.88351 | -6.887 | 5.69E-12 | *** |
| GS35:Alt1 | 0.21986 | 0.89757 | 0.245 | 0.806496 |  |
| GS40:Alt1 | 2.41131 | 0.9966 | 2.42 | 0.01554 | * |
| DS35:Alt2 | 3.9437 | 0.92535 | 4.262 | 2.03E-05 | *** |
| DS40:Alt2 | 0.22311 | 0.90055 | 0.248 | 0.804329 |  |
| GS35:Alt2 | 1.50046 | 0.91543 | 1.639 | 0.101198 |  |
| GS40:Alt2 | -0.16334 | 0.99614 | -0.164 | 0.86975 |  |
| DS35:GS35:Alt1 | 1.26423 | 1.25876 | 1.004 | 0.31521 |  |
| DS40:GS35:Alt1 | 2.36039 | 1.22776 | 1.923 | 0.054542 | . |
| DS35:GS40:Alt1 | -0.8856 | 1.41767 | -0.625 | 0.532178 |  |
| DS40:GS40:Alt1 | 9.21163 | 1.35371 | 6.805 | 1.01E-11 | *** |
| DS35:GS35:Alt2 | -2.72269 | 1.27842 | -2.13 | 0.033193 | * |
| DS40:GS35:Alt2 | -0.86452 | 1.24712 | -0.693 | 0.488178 |  |
| DS35:GS40:Alt2 | -7.86073 | 1.41239 | -5.566 | 2.61E-08 | *** |
| DS40:GS40:Alt2 | 3.59512 | 1.35039 | 2.662 | 0.007761 | ** |
